# Genetic analysis of peste des petits ruminants virus from Pakistan

**DOI:** 10.1186/1746-6148-9-60

**Published:** 2013-03-28

**Authors:** Muhammad Anees, Muhammad Zubair Shabbir, Khushi Muhammad, Jawad Nazir, Muhammad Abu Bakar Shabbir, Jonas J Wensman, Muhammad Munir

**Affiliations:** 1 Department of Microbiology, University of Veterinary and Animal Sciences, Lahore, Pakistan; 2 University Diagnostic Laboratory, University of Veterinary and Animal Sciences, Lahore, Pakistan; 3 Institute of Microbiology, University of Agriculture, Faisalabad, Pakistan; 4 Department of Clinical Sciences, Division of Ruminant Medicine and Veterinary Epidemiology, Swedish University of Agricultural Sciences, Uppsala, Sweden; 5 Department of Biomedical Sciences and Veterinary Public Health, Swedish University of Agricultural Sciences, Uppsala, Sweden

## Abstract

**Background:**

Peste des petits ruminants (PPR) is an endemic and highly contagious disease in small ruminants of Pakistan. Despite the fact that an effective vaccine is available, outbreaks are regularly occurring in the country. Thus so far, the diagnosis has primarily been made based on clinical outcome or serology. This study was carried out to characterize PPRV from an emerging wave of outbreaks from Punjab, Pakistan.

**Results:**

A total of 32 blood samples from five different flocks were tested with real-time PCR for the presence of PPRV genome. The samples detected positive in real-time PCR (n = 17) were subjected to conventional PCR for the amplification of the nucleoprotein (N) gene. Phylogenetic analysis of the sequenced N genes (n = 8) indicated the grouping of all the sequences in lineage IV along with PPRV strains from Asian and Middle East. However, interestingly sequences were divided into two groups. One group of viruses (n = 7) clustered with previously characterized Pakistani isolates whereas one strain of PPRV was distinct and clustered with Saudi Arabian and Iranian strains of PPRV.

**Conclusions:**

Results demonstrated in this study expanded the information on the genetic nature of different PPRV population circulating in small ruminants. Such information is essential to understand genetic nature of PPRV strains throughout the country. Proper understanding of these viruses will help to devise control strategies in PPRV endemic countries such as Pakistan.

## Background

Peste des petits ruminants (PPR) is a highly contagious viral disease of domestic and wild ruminants where it cause high morbidity (100%) and mortality (90%) [1,2]. The causative agent, peste des petis ruminants virus (PPRV), is grouped in the genus *Morbillivirus* within family *Paramyxoviridae* along with rinderpest, measles, phocine-, dolphin-, canine- and porpoise-distemper viruses [3]. Like other *Morbilliviruses,* PPRV is pleomorphic, with negative sense single stranded RNA genome containing 15,948 nucleotides [2,4]. The genome follows the “rule-of-six” and encodes six structural proteins that include nucleocapsid (N), phosphoprotein (P), matrix (M), fusion (F), haemagglutinin (H) and large polymerase (L) [4].

Based on the molecular characterization, strains of PPRV can be grouped into four lineages, which are genetically distinct from each other. Lineage I includes isolates from Western Africa, lineage II contains isolates from West African countries, the Ivory Coast, Guinea and Burkina Faso, and lineage III represents strains from Eastern Africa, the Sudan, Yemen and Oman [2,4-6]. The lineage IV comprises of PPRV strains from the Arabian Peninsula, the Middle East and South Asia [2,4,6]. Classification of PPRV is being analyzed based on the sequence analysis of both F and N genes; however, parallel comparison of PPRV strains has proposed that N gene is most divergent and hence most appropriate for molecular characterization of closely related isolates [7]. The virus has been recognized to occur as only one serotype among four lineages [6].

PPRV has been documented in Pakistan since 1991, however, the isolates were confirmed through PCR few years latter in 1994 [8]. Since then, PPR remained endemic in Pakistan despite use of a live attenuated vaccine in small ruminants. Due to serological monitoring facilities, it remained difficult to ascertain the level of vaccine failure and thus aggravates disease epidemiology and its control. Therefore, determining the nature of circulating strains in different parts of Pakistan is crucial to not only aid in disease diagnosis and but also to devise better control strategies in future. The present work has been conducted to determine the genetic nature of circulating PPRV strains that are continuously causing outbreaks in central Punjab, Pakistan.

## Methods

The study involves one of the livestock richest districts of Punjab where an emerging wave of clinical disease, suspected for PPRV, was reported. A brief clinical history and outcomes of different diagnostic tests are demonstrated in Table 1. The number of animals in the herd and their ages varied from 27–50 animals and 3 months-5 years, respectively. The breeds of animals were either Beetal or non-descript, without any history of vaccination in the past. Food and water were provided *ad libitum*. The morbidity and case fatality rate was in the range of 50–80% and 20–35%, respectively. A majority of infected animal died within first week of infection. Whole blood samples (n = 32) were collected aseptically from animals from five different outbreaks. The blood (200–300 μl) was poured onto QIAcard FTA Indicator Four Spots (Qiagen, Hilden, Germany), which preserve genomic material and lysed the cells and viruses.

**Table 1:** Brief history of outbreaks and outcome of different diagnostic assays

**Place of sampling in district Okara**	**Clinical findings**	**Animals sampled/total animals in the herd**	**Real-time PCR (positive/total)**	**Conventional PCR for N gene (positive/total)**
Chak 48/2-L	High fever (105–107 F), nasal discharges, sneezing, coughing, sticking of mucus to nostrils, erosions in the oral mucosa. Severe diarrhea and then drop in temperature after start of the diarrhea. Death nearly 5–10 days after start of disease	8/35	3/8	1/8
Chak 54/2-L	Fever (104-105 F), nasal discharges, sneezing, coughing, sticking of mucus to nostrils, erosive ulcerative stomatitis, diarrhea	3/27	3/3	1/3
Chak 1/4-L	Fever (104-105 F), nasal discharge, sneezing, coughing, necrotic stomatitis, severe diarrhea	9/39	5/9	2/9
Chak 31/2-L	Body temperature (105–107 F), weakness, nasal and lacrimal discharges, sneezing, coughing, erosive lesions in the oral cavity, severe diarrhea	4/50	3/4	1/4
Chak 24/2-R	Fever (104-105 F), purulent nasal discharges, coughing, erosive ulcerative stomatitis, diarrhea	8/41	3/8	3/8

The total RNA was eluted from the QIAcard FTA Indicator as described [9] and was used to screen by real-time PCR for the presence of PPRV genome as reported earlier [9,10]. All the samples appear positive in real-time PCR (Ct values <35) were used in conventional PCR to amplify N gene of PPRV for downstream sequencing [9,11]. The resultant PCR products were gel extracted and processed for sequencing using ABI PRISM BigDye Terminator version 3.1 (Applied Biosystems), according to the manufacturer’s instructions. Sequences were analyzed with an automated nucleic acid analyzer (ABI PRISM 3100; Applied Biosystems). Each DNA fragment was sequenced at least twice in both directions.

The sequences were edited and assembled using EditSeq and SeqMan suits within Lasergene 8 (version 8.0.2 13, DNASTAR, Inc., Madison, WI, USA). The resultant sequences were aligned with the sequences retrieved from GenBank representing all the lineages of PPRV. Construction of phylogenetic trees was performed with the neighbour-joining method using Kimura two-parametermodel in Mega5 version 5 (CEMI, Tempe, AZ, USA).

## Results

Out of total samples (n = 32) analyzed, real-time PCR detected only 18 samples with Ct values lower than 35. The results of real-time PCR confirmed the cause of these outbreaks to be PPRV. However, to demonstrate the genetic nature of PPRV, a conventional PCR for the amplification of N gene was performed. Although all the samples were not detected positive, a representative sample of each outbreak was amplified which appear sufficient for sequencing and downstream analysis. All the N genes were submitted to the GenBank under accession number KC207867-KC207874.

**Figure 1:**
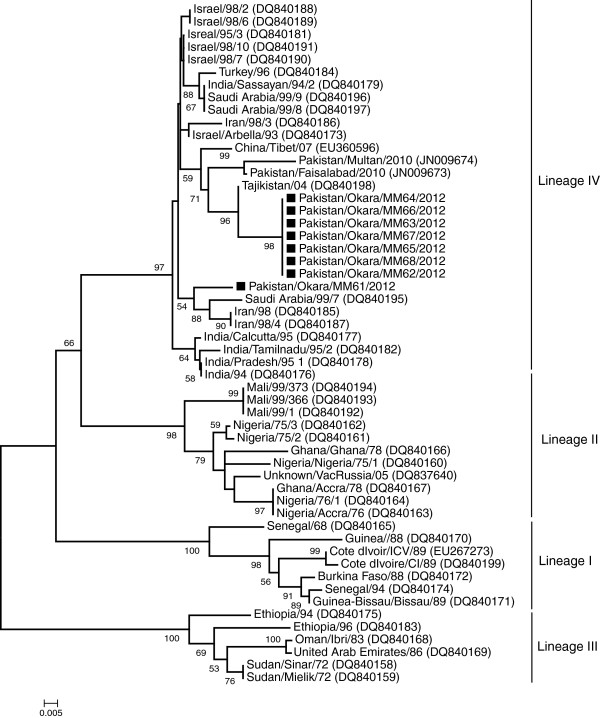
**Phylogenetic analysis of the PPRV. **Neighbor-joining majority rule consensus tree from variable N gene (255 bp) region constructed with Kimura two-parameter model in Mega5 version 5 at bootstrap value 1000 replicates. In the figure bootstrap values only above 50% are shown. The horizontal lines were proportional to the distance among sequences and the samples characterized in this study were marked with black-square (■).

Nucleotide sequence identity among eight sequences reported here and with previously characterized Pakistani strains of PPRV was found to be 97.2-100% and 94.6-97%, respectively. Phylogenetic tree, based on the 255 bp of the N genes sequenced in this study and collected from GenBank, was shown in Figure 1. All the Pakistani strains of PPRV clustered into lineage IV, which is exclusively prevalent in Asian and Middle East countries. However, it was interesting to note that all the Pakistani strains of PPRV, sequenced here or reported earlier, were grouped closed to Tajikistani strains of PPRV, except one sequence (Pakistan/Okara/MM61/2012). This strain was clustered with PPRV isolates from Saudi Arabia and Iran, and collectively made a separate cluster within lineage IV. The Pakistan/Okara/MM61/2012 strain carried eight amino acid differences from rest of the strains studied here. Taken together, the results demonstrated that there are at least two different population of PPRV circulating in the country.

## Discussion

Since first report of PPRV in Pakistan, several outbreaks have been reported [2,9,12-19]. Most of these were based on either clinical history, antigen or antibody detection. However, molecular characterization of the virus is considered a key to understand the genetics of the causative agents. The results of real-time and conventional PCR from blood samples, transported onto FTA cards, confirmed the fact that they are suitable, reliable and economical mean of transportation [9,19], especially where maintenance of cold chain is not possible or legislations for shipment of biological material is complicated [2]. Conventional and real-time PCR for the detection of PPRV have shown variable sensitivity and specificity. Therefore these tests must be evaluated in parallel before their implementations for routine diagnostic purposes in specific laboratory settings.

**Figure 2:**
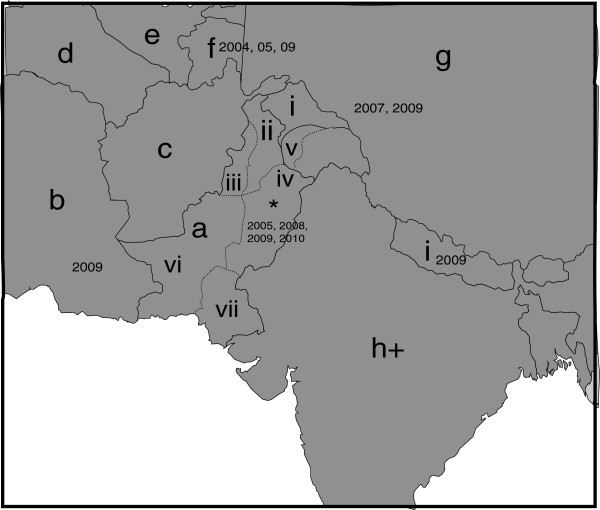
**Detection of PPRV in Pakistan and surrounding countries. a**) Pakistan; **b**) Iran; **c**) Afghanistan; **d**) Turkmenistan; **e**) Uzbekistan; **f**) Tajikistan; **g**) China; **h**) India; **i**) Nepal. Dates shown on the map illustrate both published reports and reports to the OIE reference laboratories as detailed in Banyard et al., 2010 (20). For Pakistan, provinces are shown: *i*) Gilgit baltistan/Northern Areas; *ii*) Khyber pakhtunkhwa; *iii*) Tribal areas; *iv*) Punjab; *v*) Azad Jammu & Kashmir; *vi*) Baluchistan; *vii*) Sindh province. The approximate position of the outbreak detailed in this study is represented by a star. + India frequently reports outbreaks as PPRV is endemic across the country (20).

Latest data has shown that N gene reveals improved image of PPRV epidemiology and is considered better than F gene based distribution of PPRV strains [6,20]. As this fact is well established that PPRV has propensity to change genetically, N gene based phylogenetic analysis was performed in this study. The similarity and divergence among samples from five outbreaks and formerly characterized PPRV isolates were evaluated by analyzing their common N gene sequences. In general, the topology of the tree revealed PPRV isolates from diverse geographical areas differ largely in their partial N gene sequence. The tree shows that currently analyzed PPRV are more closely related to Tajikistan. The possible relationship of Pakistani strains to that of Tajikistan strains of PPRV could be due to the fact that Pakistan with its province named Gilgit Baltistan shares the Pamir region with Tajikistan through Wakhan corridor with Afghanistan where animal movement in the mountainous grazing lands of Pamir region is not uncommon between these countries (Figure 2). Moreover, selling of small ruminants by nomads in Gilgit Baltistan province to rest of the country, on or before religious ceremonies such as Eid-ul-Adha, is common particularly in Punjab province that represents about 60% of total population. The grouping of these strains of PPRV at two different places in the tree shows that PPRV isolates may have undergone exceptional evolutionary pathway or may be a result of several introduction of viruses from a variety of sources. However, such interpretations required confirmation by the analysis of larger samples collected from wide range of outbreaks.

## Conclusions

In conclusion, lineage IV of PPRV is currently circulating in the country, with certain level of genetic diversity. Since serological assays (i.e. cELISA) don’t necessary indicate the current persistence of the infection, it is essential to implement molecular diagnosis along with characterization. Despite PPRV is endemic in Pakistan and outbreaks are regularly occurring, limited information is available on the genetic nature of PPRV. The sequences reported here expand the available information on the circulating strains of PPRV in Pakistan. There is no sequence information for any of the N genes of PPRV is available other than our previously characterized sequences and the sequences reported here. With these limited data, we still manage to demonstrate the presence of two different population of PPRV, which warrant future need to perform such studies at country level to ascertain the complete picture of circulating viruses in the country. Such understating is crucial for devising future control plans. Currently, vaccination is recommended in certain areas of the country. This vaccination is based on Nig75/1, which belong to lineage II, while field isolates from Pakistan are grouped in lineage IV. Genetic characterization of field strains will provide foundations for construction of vaccines from domestic strains as has recently been practiced in India.

## Abbreviations

PPR: Peste des petits ruminants; N: Nucleocapsid; P: Phosphoprotein; M: Matrix; F: Fusion; H: Haemagglutinin; L: Large polymerase; PCR: Polymerase chain reaction; cELISA: Competitive enzyme linked immunosorbant assay.

## Competing interests

The authors declare that they have no competing interests.

## Authors’ contributions

MM, MA, MZS designed the study, MM conducted the experiment, MZS, MA KM, JN, MABS, MM wrote the paper with input from all authors. All authors read and approved the final manuscript.
